# The evaluation of the effect of mindfulness and metacognition on anxiety symptoms: A case-control study

**DOI:** 10.1192/j.eurpsy.2022.998

**Published:** 2022-09-01

**Authors:** O. Aydın, F. Obuca, E. Çakıroğlu, P. Ünal-Aydın, A. Esen-Danacı

**Affiliations:** 1 International University of Sarajevo, Psychology, Ilidza, Bosnia and Herzegovina; 2 Celal Bayar University, Psychiatry, Manisa, Turkey

**Keywords:** Anxiety, Mindfulness, metacognition, metacognitive beliefs

## Abstract

**Introduction:**

Anxiety disorders (ADs) are pervasive, detrimental, and associated with numerous psychiatric disorders; however, their etiology and effective treatment strategies are not yet fully explored.

**Objectives:**

We aimed to study whether the symptom severity of ADs is related to mindfulness and metacognition among adults. In addition, we wanted to compare metacognition and mindfulness between patients with ADs and healthy controls (HC).

**Methods:**

Two hundred participants were enrolled in this study. Structured clinical interview, sociodemographic form, Five Facet Mindfulness Questionnaire-Short Form (FFMQ-S), Metacognition Questionnaire-30 (MCQ-30), and Hamilton Anxiety Rating Scale (HAM-A) were administered. Multivariate analysis of covariance (MANCOVA) was conducted to compare the groups in terms of mindfulness and metacognition. Correlation and multiple linear regression analyses were performed to measure the association between anxiety symptom severity, mindfulness, and metacognition.

**Results:**

The main finding indicates that Positive Beliefs about Worry are associated with reduced symptom severity of ADs. Furthermore, the results suggest that HC have more Positive Beliefs about Worry and Nonjudging of Inner Experience compared to patients with ADs, who use Negative Beliefs about Uncontrollability and Danger and Need to Control Thoughts to a greater extent.

**Conclusions:**

This study suggests that dysfunctional metacognitive beliefs may influence symptom severity of ADs among adults. We advise that focusing on reducing maladaptive metacognitions may be beneficial while treating ADs in adults

**Disclosure:**

No significant relationships.

